# The impact of pulmonary embolism on health outcomes of COVID-19 at 3 months after hospitalization

**DOI:** 10.1016/j.rpth.2024.102573

**Published:** 2024-09-16

**Authors:** Chantal Visser, Julia C. Berentschot, Cindy M.M. de Jong, M. Louisa Antoni, L. Martine Bek, Rita J.G. van den Berg-Emons, Bram van den Borst, Hugo ten Cate, Arina J. ten Cate-Hoek, Dionne C.W. Braeken, J.J. Miranda Geelhoed, Majanka H. Heijenbrok-Kal, Sander M.J. van Kuijk, Lucia J.M. Kroft, Jenneke Leentjens, Anna H.E. Roukens, Suzanne C. Cannegieter, Frederikus A. Klok, Marieke J.H.A. Kruip, Merel E. Hellemons, Dutch COVID & Thrombosis Coalition@CovidDutch

**Affiliations:** 1 Department of Hematology, Erasmus MC, Erasmus University Medical Center Rotterdam, Rotterdam, the Netherlands; 2 Department of Respiratory Medicine, Erasmus MC, Erasmus University Medical Center Rotterdam, Rotterdam, the Netherlands; 3 Department of Medicine - Thrombosis and Hemostasis, Leiden University Medical Center, Leiden, the Netherlands; 4 Department of Cardiology, Heart Lung Centre, Leiden University Medical Center, Leiden, the Netherlands; 5 Department of Rehabilitation Medicine, Erasmus MC, Erasmus University Medical Center Rotterdam, Rotterdam, the Netherlands; 6 Department of Pulmonary Diseases, Radboud University Medical Center, Nijmegen, the Netherlands; 7 Department of Internal Medicine, Division of Vascular Medicine, Maastricht University Medical Center (MUMC+), Maastricht, the Netherlands; 8 Cardiovascular Research Institute Maastricht (CARIM), Maastricht, the Netherlands; 9 Department of Biochemistry, Laboratory for Clinical Thrombosis and Hemostasis, Maastricht University, Maastricht, the Netherlands; 10 Thrombosis Expertise Center, Maastricht University Medical Center (MUMC+), Maastricht, the Netherlands; 11 Department of Pulmonary Diseases, Leiden University Medical Center, Leiden, the Netherlands; 12 Department of Clinical Epidemiology and Medical Technology Assessment, Maastricht University Medical Center (MUMC+), Maastricht, the Netherlands; 13 Department of Radiology, Leiden University Medical Center, Leiden, the Netherlands; 14 Department of Internal Medicine, Radboud University Medical Center, Radboud University, Nijmegen, the Netherlands; 15 Department of Infectious Disease, Leiden University Medical Center, Leiden, the Netherlands; 16 Department of Clinical Epidemiology, Leiden University Medical Center, Leiden, the Netherlands

**Keywords:** COVID-19, patient reported outcome measures, pulmonary embolism, quality of life, respiratory function tests

## Abstract

**Background:**

COVID-19 patients frequently experience pulmonary embolism (PE), but its long-term consequences remain uncertain.

**Objectives:**

To assess the impact of PE in COVID-19 patients on health outcomes at 3 months after hospitalization.

**Methods:**

In this multicenter cross-sectional study, we aggregated data from existing databases to evaluate the impact of PE on health outcomes at 3 months after hospitalization. We assessed 1) questionnaires on health-related quality of life (5-level EuroQol 5-dimensional questionnaire [EQ-5D-5L] questionnaire), anxiety, depression, cognitive failure, and posttraumatic stress disorder; 2) pulmonary function tests (diffusing capacity of the lungs for carbon monoxide [DLCO] and spirometry); and 3) radiological abnormalities. We developed 3 models to assess the association between PE and the EQ-5D-5L index and the percentage of predicted DLCO (DLCO%): a crude model (model 1), adjusted for age, sex, and presence of comorbidities (model 2), and model 2 additionally adjusted for intensive care unit admission (model 3).

**Results:**

We included 465 patients who had been hospitalized for COVID-19, of whom 102 (21.9%) had developed a PE during admission. Patients with PE had poorer EQ-5D-5L index values, more impairment in pulmonary functions, and more frequent radiological abnormalities than patients without PE. Symptoms of anxiety, depression, cognitive failure, and posttraumatic stress disorder did not differ between the 2 groups. In model 2, PE was associated with lower EQ-5D-5L index and lower DLCO%. After additionally adjusting for intensive care unit admission, the association between PE and lower EQ-5D-5L index (mean difference = −0.069, [95% CI, −0.12 to −0.017]) remained but not between PE and DLCO%.

**Conclusion:**

Our findings suggest that PE in COVID-19 patients is associated with reduced health-related quality of life at 3 months after hospitalization. While PE may be a marker of COVID-19 severity, its presence during hospitalization could indicate potential long-term health issues, which may be considered during follow-up care.

## Essentials

• The clinical impact of pulmonary embolism (PE) in COVID-19 patients remains unclear.
• This multicenter study assessed health outcomes 3 months after discharge in COVID-19 patients.
• Patients with PE had lower quality of life and impaired lung function 3 months after discharge.
• PE may be a marker of COVID-19 severity and could indicate potential long-term health issues.

## 1 Introduction

Hospitalized COVID-19 patients are at risk of thrombotic complications, with acute pulmonary embolism (PE) as the most frequently occurring [1,2]. A meta-analysis has shown a high incidence of PE in hospitalized COVID-19 patients, with an incidence of 17% in nonintensive care unit (ICU) patients and 26% in ICU patients [2]. While previous studies have focused on predictors for PE and acute clinical outcomes in COVID-19 patients with diagnosed PE [3,4], there is limited data on the long-term impact of PE on health outcomes in this population [5].

In non–COVID-19 patients, PE survivors often experience long-term health sequelae. These include new-onset or increased dyspnea, functional impairment, and decreased cardiopulmonary reserve, and are reported in up to 50% of PE survivors [[6], [7], [8]]. This so-called post-PE syndrome is associated with increased frailty, symptoms of anxiety and depression, and reduced health-related quality of life (HRQoL) [[8], [9], [10], [11], [12]]. In severe, but rare, cases, chronic thromboembolic pulmonary hypertension (CTEPH) may develop, leading to a considerable loss of HRQoL and increased mortality [13].

Many patients hospitalized for COVID-19 experience long-term health effects and different terms have been used to describe this condition, such as “Post-Acute COVID-19 Syndrome,” “Long COVID,” and “post–COVID-19 condition.” Fatigue and neurocognitive impairment are frequently reported health problems after hospitalization for COVID-19 [14,15]. Moreover, COVID-19 patients may experience reduced HRQoL [16,17].

Based on these considerations, we aimed to assess various health outcomes in patients with and without PE, including patient-reported outcome measures (PROMs), pulmonary function, and radiological abnormalities, 3 months after hospitalization for COVID-19. The primary outcomes were HRQoL and diffusing capacity of the lungs for carbon monoxide (DLCO), other outcomes were considered as secondary outcomes. These primary outcomes were commonly found to be reduced in both non–COVID-19 patients with PE and COVID-19 patients [[8], [9], [10],^12^,^16^,[18], [19], [20], [21], [22], [23]]. Furthermore, we conducted multiple multivariable regression analyses to explore the association between PE and both primary and secondary outcomes.

## 2 Methods

### 2.1 Study design and participants

This multicenter observational study was conducted across 4 Dutch academic hospitals: the Erasmus University Medical Center (UMC), Leiden UMC, Maastricht UMC, and Radboud UMC. We retrospectively collected and combined data from pre-existing cohorts of COVID-19 patients in the participating hospitals, as described in detail elsewhere [[24], [25], [26], [27], [28]], and performed a cross-sectional analysis at 3 months after discharge. These databases had been established as part of routine follow-up procedures or local study procedures; for a detailed description, see Supplementary Material S1. During the first COVID-19 wave in the Netherlands, the participating academic hospitals routinely offered follow-up around 3 months after discharge. As part of this 3-month visit, patients performed pulmonary function tests, chest imaging, and questionnaires, which we used in the current study.

Eligible patients had been discharged before September 2020 and of whom data were available from at least one of the following: pulmonary function tests, chest computed tomography (CT) scans, or one or more PROMs using questionnaires, all performed as part of the 3-month visit. Ethical approval was obtained from each participating site, and either informed consent had been obtained or an opt-out procedure had been applied. The current study was approved by the Erasmus UMC (MEC-2021-0423).

### 2.2 Data collection

Demographic (age, sex, body mass index, and smoking status) and clinical (comorbidities, laboratory values, diagnosis of PE, chest CT scan abnormalities, admission to ICU, invasive mechanical ventilation, number of days in ICU, number of days in the hospital, and treatment for COVID-19) characteristics during hospital admission were collected from the databases of the pre-existing cohorts using a standardized format provided to the local researchers of the participating hospitals. In these databases, sex was defined based on the information recorded in the electronic patient record, which categorized individuals as male or female, primarily according to their registered biological characteristics. If variables were missing from these databases, local researchers retrospectively collected this information from the electronic patient records. Patients who were classified as having had PE during hospitalization were those who displayed suspected thrombotic complications, and in whom the diagnosis was verified through CT pulmonary angiography (CTa) or clinical assessment. Clinical assessment encompassed PE diagnosis in cases where CTa was omitted due to patients’ inability to be transferred to the CT scan. PE was categorized based on the most proximal location in the pulmonary arteries (central, segmental, or subsegmental). All other patients were classified as not having had PE. Data had been deidentified and stored in separate databases, which were merged for this study.

### 2.3 Outcomes

We evaluated HRQoL and DLCO as primary outcomes, while other PROMs, spirometry, and chest radiological abnormalities were considered secondary outcomes.

#### 2.3.1 PROMs

We used PROMs that had been assessed in at least 2 of the participating hospitals. When the same health outcome was assessed by different questionnaires, we used validated cut-off scores to indicate the presence of a specific health complaint and combined the dichotomous outcome of the questionnaires.

HRQoL was assessed with the 5-level EuroQol 5-dimensional questionnaire (EQ-5D-5L) across all hospitals and consists of 5 domains (mobility, self-care, usual activities, pain/discomfort, and anxiety/depression) [29]. The EQ-5D-5L dimension scores were converted into a single index value ranging from less than 0 (negative values indicating a health state worse than death) to 1 (representing perfect health). The EQ-5D-5L also includes a vertical visual analog scale (VAS) to assess self-rated general health status on a scale from 0 (worst imaginable health status) to 100 (best imaginable health status). We used age- and sex-adjusted median index values obtained from the Dutch tariff for the EQ-5D-5L as reference values [30].

We also assessed symptoms of anxiety, depression, PTSD, and cognitive failures. Symptoms of anxiety were assessed with the Hospital Anxiety and Depression Scale [31] (HADS) subscale anxiety (HADS-A) and Generalized Anxiety Disorder (GAD-7) [32] questionnaires. Validated cut-off scores of ≥8 for HADS-A and ≥10 for GAD-7 were used to indicate signs of anxiety. Symptoms of depression were assessed with the HADS subscale for depression (HADS-D) [31] and the Patient Health Questionnaire-9 (PHQ-9) [33], with cut-off scores of ≥8 for the HADS-D and ≥10 for the PHQ-9 indicating depression. Symptoms of PTSD were assessed with the PTSD Checklist for DSM-5 [34] (PCL-5) and the Impact of Event Scale-Revised (IES-R) [35]; in both questionnaires, scores ≥33 indicate clinically significant PTSD. Cognitive failures were assessed using the Cognitive Failure Questionnaire (CFQ), with scores >43 indicating cognitive failure [36,37].

#### 2.3.2 Pulmonary function

Pulmonary function tests included the assessment of diffusing capacity of the lungs for carbon monoxide (DLCO) in mmol·min^−1^·kPa^−1^ and spirometry to assess forced vital capacity (FVC) and forced expiratory volume in 1 second (FEV_1_) in liters, following the guidelines of the American Thoracic Society and the European Respiratory Society [38]. Pulmonary function outcomes are also presented as a percentage of predicted DLCO (DLCO%), FVC (FVC%), and FEV_1_ (FEV_1_%) values, using references values from the Global Lung Function Initiative Network [39,40]. Abnormal values were defined as those below the lower limit of normal (<LLN; z-score below −1.64).

#### 2.3.3 Radiological abnormalities

Chest CT scans were assessed by experienced radiologists using a standardized assessment. We used the radiological reports to score the presence of distinctive parenchymal abnormalities across 5 categories: ground-glass opacities (GGOs), subpleural lines/bands, bronchiectasis, reticulation/fibrosis, and consolidation.

### 2.4 Statistical analysis

Continuous variables are presented as mean (SD) or as median (IQR). Categorical variables are presented as count (percentage). The normality of variables was assessed using histograms. We compared health outcomes at follow-up between COVID-19 patients with and without PE using 2-sample *t*-test or Mann-Whitney *U* test for continuous variables and Fisher’s exact test for dichotomous variables. The median difference and 95% CI between the EQ-5D-5L index and age- and sex-adjusted median Dutch Tariff index values were calculated using the Bonett and Price method, as the distribution of the median Dutch Tariff index values was skewed [41]. *P* values are corrected for multiple comparisons using Bonferroni correction.

Multiple multivariable linear regression analysis and logistic regression analysis for symptoms of anxiety and depression were performed to assess the association between PE and the primary outcomes EQ-5D-5L index and DLCO%, and secondary outcomes including PROMs (anxiety, depression, and cognitive failures) and spirometry (FEV_1_ and FVC). A directed acyclic graph was used to identify potential confounders and mediators (Supplementary Material S2 and Supplementary Figure). Three models were explored for each outcome: 1) a crude model containing only PE (model 1); 2) model 1 with age at admission, sex, and the presence of any comorbidity (diabetes mellitus, cardiovascular disease, chronic kidney disease, chronic liver disease, chronic lung disease, pre–COVID-19 VTE, stroke, active cancer, immunodeficiency or hypertension) as confounders. For DLCO%, we replaced the covariate of any comorbidity with the presence of chronic lung disease; 3) model 2 plus the addition of ICU admission.

We tested the additivity assumption by comparing the complex model, including *a priori* considered interactions, to the model without these interactions using a likelihood ratio test. Multiplicative interactions were added to the final model and separate analyses were performed when the interaction was significant. We used a *P* value threshold for significance of the interaction term of 0.15. Second, the linearity of the relationship between the health outcomes and age was examined using natural splines (splines package, R) [42] and incorporated into the final model if applicable. Results of the multivariable regression analyses are expressed as mean difference (MD) or odds ratio (OR) with 95% CI.

We performed 2 explorative subgroup analyses and one sensitivity analysis. We performed multivariable linear regression analysis to calculate the MD in EQ-5D-5L index and DLCO% for different patient groups. First, regarding potential associations of PE and ICU admission, patients were categorized into admitted to the ICU with (group 1, reference group) and without PE (group 2) and patients without ICU admission with (group 3) and without PE (group 4) and adjusted for sex and age at admission. Second, regarding PE severity, patients were categorized based on the most proximal location of PE: patients without PE (group 1, reference group), patients with subsegmental PE (group 2), patients with segmental or central PE (group 3), and patients with PE based on clinical assessment (group 4). Finally, a sensitivity analysis was performed in which patients with PE based on clinical assessment were excluded. Statistical analyses were performed using IBM SPSS Statistics (v.28) and R software (v.4.2.1) [42].

## 3 Results

In total, 465 patients who had been hospitalized for COVID-19 and completed at least one of the outcome measurements at the 3-month follow-up visit were identified. Of those, 102 (21.9%) patients had been diagnosed with PE during hospitalization. The localization of PE was most often segmental (43/102, 42.5%), thereafter subsegmental (40/102, 39.2%), central (11/102, 10.8%), and location unknown or clinically diagnosed (8/102, 7.8%). The median age of patients with PE was 59.5 (IQR, 54.0-66.0) years and 77 (75.5%) were male. Among patients without PE, the median age was 60.0 (53.0-69.0) years and 236 (65.0%) were male. Other characteristics observed at hospital admission are summarized in Table 1. The median follow-up time (days between hospital discharge and follow-up assessment) was similar between patients with PE (85.5 [68.0-102.8] days) and those without (87.0 [68.0-102.0] days).

**Table 1:** Characteristics of participants enrolled in the study.

Characteristics at baseline	*n*	Total (*n* = 465)	No PE (*n* = 363)	PE (*n* = 102)
**Demographics**				
Age (y)	465	60.3 ± 12.2	60.5 ± 12.5	59.4 ± 11.0
Male sex	465	313 (67.3)	236 (65.0)	77 (75.5)
BMI (kg/m^2^)	414	27.7 (24.9-31.2)	27.8 (24.9-31.7)	27.7 (24.9-30.3)
Smoking status	430			
Never		207 (48.1)	159 (47.2)	48 (51.6)
Former		217 (50.5)	172 (51.0)	45 (48.4)
Current		6 (1.4)	6 (1.8)	0 (0.0)
**Medical history**				
No comorbidity	464	106 (22.8)	77 (21.3)	29 (28.4)
Diabetes mellitus	426	69 (16.2)	55 (16.1)	14 (16.7)
Cardiovascular disease	426	160 (37.6)	133 (38.9)	27 (32.1)
Chronic kidney disease	426	28 (6.6)	26 (7.6)	2 (2.4)
Chronic liver disease	465	8 (1.7)	7 (1.9)	1 (1.0)
Chronic lung disease	426	91 (21.4)	80 (23.4)	11 (13.1)
Pre–COVID-19 VTE	462	18 (3.9)	13 (3.6)	5 (5.0)
Stroke	462	17 (3.7)	16 (4.4)	1 (1.0)
Active cancer	423	40 (9.5)	35 (10.3)	5 (6.0)
Immunodeficiency	423	41 (9.7)	37 (10.9)	4 (4.8)
**In-hospital characteristics**				
Laboratory values				
LDH (U/L)	456	366.0 (292.0-473.0)	352.0 (280.5-451.0)	415.0 (335.0-530.0)
Ferritin (μg/L)	318	926.0 (475.8-1686.8)	839.0 (422.0-1671.5)	1269.0 (584.0-1910.0)
D-dimer (mg/L)	253	1.2 (0.7-2.5)	1.1 (0.7-1.9)	2.0 (1.0-5.8)
Chest CT scan abnormalities				
No abnormalities	394	9 (2.3)	7 (2.3)	2 (2.2)
Ground-glass opacities	391	373 (95.4)	283 (95.0)	93 (96.8)
Consolidations	392	266 (67.9)	201 (67.2)	65 (69.9)
Reticulation/fibrosis	321	64 (19.9)	54 (22.5)	10 (12.3)
Bronchiectasis	306	41 (13.4)	30 (13.3)	11 (13.8)
Curvilinear bands	299	36 (12.0)	25 (11.4)	11 (13.9)
Requiring oxygen supplementation	461	410 (88.9)	317 (88.1)	93 (92.1)
ICU admission	465	217 (46.7)	133 (36.6)	84 (82.4)
Invasive mechanical ventilation	465	199 (42.8)	116 (32.0)	83 (81.4)
Extracorporeal membrane oxygenation	465	5 (1.1)	3 (0.8)	2 (2.0)
Length of ICU stay, d	216	21.0 (12.0-33.8)	16.5 (8.3-29.0)	28.5 (18.0-40.0)
Length of hospital stay, d	347	9.0 (5.0-26.0)	8.0 (4.0-16.0)	32.0 (20.0-46.5)
Treatment				
Hydroxychloroquine	463	228 (49.2)	185 (51.1)	43 (42.6)
Corticosteroids	461	126 (27.3)	86 (23.8)	40 (40.4)
Antivirals	463	56 (12.1)	40 (11.0)	16 (15.8)
Antibiotic agent	460	351 (76.3)	264 (73.3)	87 (87.0)
Antifungal agent	460	124 (27.0)	69 (19.2)	55 (55.0)
Antiplatelet therapy^a^	460	75 (16.3)	61 (17.0)	14 (13.9)

Data are presented as mean with ±SD, median with IQR or as a number with percentage.

BMI, body mass index; CT, computed tomography; ICU, intensive care unit; LDH, lactate dehydrogenase; PE, pulmonary embolism; VTE, venous thromboembolism.

a Antiplatelet therapy comprises patients in whom antiplatelet therapy was initiated during hospitalization and patients with chronic antiplatelet therapy that was continued during hospital admission.

### 3.1 PROMs

**Figure 1:**
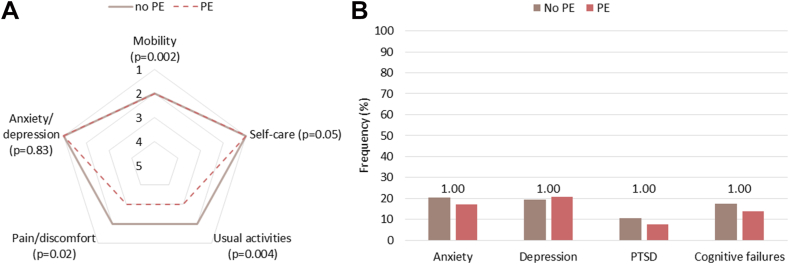
Patient-reported outcomes in COVID-19 patients with and without pulmonary embolism during hospitalization at 3 months follow-up. Patient-reported outcome measures were assessed around 3 months after hospital discharge. (A) Median group scores across the dimensions of the 5-level EuroQol 5-dimensional questionnaire. Each dimension has 5 answer levels: no problems (1), some problems (2), moderate problems (3), severe problems (4), and extreme problems/unable to (5). For categorical outcomes for each domain, see Supplementary Table S1. Group differences in 5-level EuroQol 5-dimensional questionnaire scores were assessed using a Mann-Whitney *U* test and *P* values are presented, using Bonferroni corrected *P* values for multiple testing. (B) Frequency of patients with symptoms of anxiety, depression, posttraumatic stress disorder (PTSD), and cognitive failures. Symptoms of anxiety were indicated by scores ≥8 on the anxiety subscale of the Hospital Anxiety and Depression Scale or scores ≥10 on the Generalized Anxiety Disorder questionnaire. Symptoms of depression were indicated by scores ≥8 on the depression subscale of the Hospital Anxiety and Depression Scale or scores ≥10 on the Patient Health Questionnaire-9. Symptoms of PTSD were indicated by scores ≥33 on the PTSD Checklist for DSM-5 (cut-off ≥33) or scores ≥33 on the Impact of Event Scale-Revised. Subjective cognitive failures in everyday life were indicated by scores >43 on the Cognitive Failure Questionnaire. Group differences for symptoms of anxiety, depression, PTSD, and cognitive failures were assessed using Fisher’s exact test and *P* values are presented in the figure, using Bonferroni corrected *P* values for multiple testing. PTSD, posttraumatic stress disorder.

In total, 405 (87.1%) patients completed at least one questionnaire at follow-up. The EQ-5D-5L dimension scores are presented per group in Figure 1A, for categorical outcomes see Supplementary Table S1. Patients with PE had a lower mean EQ-5D-5L index value than patients without PE (Tables 2 and 3) (MD: −0.086 [95% CI: −0.13 to −0.038]). Both groups, those with PE (median difference: −0.18 [95% CI: −0.18 to −0.18]) and those without PE (median difference: −0.09 [95% CI: −0.09 to −0.09]) exhibited lower index values compared with age- and sex-adjusted Dutch Tariff median index values, with patients with PE showing a more pronounced difference. Regarding other PROMs, symptoms of anxiety, depression, PTSD, and cognitive failures did not differ between patients with and without PE (Figure 1B and Table 2).

**Table 2:** Patient-reported outcome measures, pulmonary function, and chest radiology abnormalities in COVID-19 patients with and without pulmonary embolism (reference) during hospitalization at 3 months follow-up.

Health outcomes at 3 months post-hospitalization	*n*	Total (*n* = 465)	*n*	No PE (*n* = 363)	*n*	PE (*n* = 102)	MD/OR (95% CI)
**PROMs**							
HRQoL, *n*	318		236		82		
EQ-5D-5L, index value		0.74 ± 0.19		0.76 ± 0.19		0.67 ± 0.20	MD: –0.086 (–0.13 to –0.038)
EQ-5D-5L, VAS		65.8 ± 18.6		67.4 ± 18.7		61.3 ± 17.7	MD: –6.1 (–1.6 to –10.6)
Anxiety, *n*	380		298		82		
HADS-A, total score		4.8 ± 4.1		4.8 ± 4.0		4.9 ± 4.4	MD: 0.09 (–1.1 to 1.3)
GAD7, total score		3.4 ± 3.9		3.4 ± 4.3		3.4 ± 3.1	MD: –0.004 (–2.0 to 2.0)
Anxiety (HADS-A ≥ 8 or GAD7 ≥ 10)		75 (19.7)		61 (20.5)		14 (17.1)	OR: 0.8 (0.4 to 1.5)
Depression, *n*	384		302		82		
HADS-D, total score		4.6 ± 3.9		4.4 ± 3.8		5.0 ± 4.3	MD: 0.5 (–0.6 to 1.7)
PHQ9, total score		5.1 ± 5.1		5.2 ± 5.1		4.7 ± 5.2	MD: –0.5 (–3.6 to 2.6)
Depression (HADS-D ≥ 8 or PHQ9 ≥ 10)		79 (20.6)		63 (20.9)		16 (19.5)	OR: 0.9 (0.5 to 1.7)
PTSD, *n*	261		197		64		
PCL5, total score		12.1 ± 12.9		12.6 ± 13.3		10.4 ± 11.5	MD: –2.1 (–6.1 to 1.8)
IES-R, total score		16.1 ± 14.8		16.1 ± 14.5		16.1 ± 15.7	MD: 0.005 (–9.2 to 9.2)
PTSD (PCL5 ≥ 33 or IES-R ≥ 33)		26 (10.0)		21 (10.7)		5 (7.8)	OR: 0.7 (0.2 to 2.1)
Cognitive functioning, n	254		189		65		
CFQ total score		27.7 ± 16.8		28.0 ± 16.6		26.7 ± 17.5	MD: –1.3 (–6.2 to 3.6)
Cognitive failure (CFQ > 43)		42 (16.5)		33 (17.5)		9 (13.8)	OR: 0.8 (0.3 to 1.8)
**Radiological abnormalities**							
Chest CT scan abnormalities, *n*							
No abnormalities	376	30 (8.0)	284	29 (10.2)	92	1 (1.1)	OR: 0.1 (0.002 to 0.6)
Ground to glass opacities	392	269 (68.6)	299	195 (65.2)	93	74 (79.6)	OR: 2.1 (1.2 to 3.8)
Subpleural curvilinear lines/bands	366	184 (50.3)	275	125 (45.5)	91	59 (64.8)	OR: 2.2 (1.3 to 3.7)
Bronchiectasis	366	153 (41.8)	277	99 (35.7)	89	54 (60.7)	OR: 2.8 (1.7 to 4.7)
Reticulation/fibrosis	341	112 (32.8)	261	80 (30.7)	80	32 (40.0)	OR: 1.5 (0.9 to 2.6)
Consolidations	390	47 (12.1)	298	28 (9.4)	92	19 (20.7)	OR: 2.5 (1.2 to 4.9)
**Pulmonary function**							
Spirometry							
FVC, L	393	3.9 ± 1.1	297	3.9 ± 1.1	96	3.8 ± 1.1	MD: –0.2 (–0.4 to 0.1)
FVC, % predicted	394	93.1 ± 19.1	298	94.8 ± 19.6	96	88.0 ± 16.7	MD: –6.8 (–11.2 to –2.4)
FVC<LLN, *n* (%)	385	72 (18.7)	290	46 (15.9)	95	26 (27.4)	OR: 2.0 (1.1 to 3.6)
FEV_1_, L	398	3.0 ± 0.8	300	3.0 ± 0.8	98	3.0 ± 0.8	MD: –0.08 (–0.3 to 0.1)
FEV_1_, % predicted	399	92.7 ± 18.8	301	94.0 ± 19.3	98	88.7 ± 16.6	MD: –5.3 (–9.5 to –1.0)
FEV_1_<LLN, *n* (%)	390	66 (16.9)	293	45 (15.4)	97	21 (21.6)	OR: 1.5 (0.8 to 2.8)
Gas exchange							
DLCO, mmol·min^−1^·kPa^−1^	369	6.6 ± 2.1	281	6.7 ± 2.1	88	6.2 ± 2.1	MD: –0.5 (–1.0 to –0.01)
DLCO, % predicted	368	75.0 ± 18.9	280	76.9 ± 18.7	88	69.0 ± 18.2	MD: –7.9 (–12.4 to –3.5)
DLCO < LLN, *n* (%)	368	183 (49.7)	279	124 (44.4)	89	59 (66.3)	OR: 2.5 (1.5 to 4.2)

Data are presented as mean with ±SD for continuous variables and as a number with percentage for categorical variables.

PROM, patient-reported outcome measure; PE, pulmonary embolism; MD, mean difference; OR, odds ratio; FVC, forced vital capacity; LLN, lower limit of normal; FEV_1_, forced expiratory volume in 1 second; DLCO, diffusing lung capacity for carbon monoxide; CT, computed tomography; HRQoL, Health-Related Quality of Life; EQ-5D-5L; 5-level EuroQol 5D questionnaire; VAS, visual analog scale; HADS, Hospital Anxiety and Depression Scale; HADS-A, Hospital Anxiety and Depression Scale with the subscale anxiety; HADS-D, Hospital Anxiety and Depression Scale with the subscale depression; GAD7, General Anxiety Disorder-7; PHQ9, Patient Health Questionnaire-9; PTSD, posttraumatic stress disorder; PCL5, PTSD Checklist for DSM-5; IES-R, Impact of Event Scale-Revised; CFQ, Cognitive Failure Questionnaire.

**Table 3:** Mean difference or odds ratio in primary and secondary outcome measures between COVID-19 patients with and without pulmonary embolism (reference) during hospitalization at 3 months follow-up.

Health outcomes at 3 months post-hospitalization	Total (*n* = 465)	No PE (*n* = 363)	PE (*n* = 102)	Crude model (model 1)	Model 2^a^	Model 3^b^
**Primary outcome measures**						
EQ-5D-5L index value	0.74 ± 0.19	0.76 ± 0.19	0.67 ± 0.20	MD: −0.086 (−0.13 to −0.038)	MD: −0.095 (−0.14 to −0.047)	MD: −0.069 (−0.12 to −0.017)
DLCO (% predicted)^c^	75.0 ± 18.9	76.9 ± 18.7	69.0 ± 18.2	MD: −7.92 (−12.38 to −3.45)	MD: −8.09 (−12.48 to −3.70)	MD: −2.21 (−6.61 to 2.19)
**Secondary outcome measures**						
Anxiety (HADS-A ≥ 8 or GAD7 ≥ 10)	75 (19.7%)	61 (20.5%)	14 (17.1%)	OR: 0.80 (0.41 to 1.48)	OR: 0.84 (0.43 to 1.58)	OR: 0.71 (0.34 to 1.42)
Depression (HADS-D ≥ 8 or PHQ9 ≥ 10)	79 (20.6%)	63 (20.9%)	16 (19.5%)	OR: 0.92 (0.48 to 1.67)	OR: 0.98 (0.51 to 1.80)	OR: 0.94 (0.46 to 1.85)
Total CFQ score^c^^,^^d^	27.6 ± 16.8	28.0 ± 16.6	26.7 ± 17.5	MD: −1.30 (−6.17 to 3.57)	MD: −1.00 (−5.61 to 3.60)	MD: −0.66 (−5.85 to 4.53)
FVC (% predicted)^c^	93.1 ± 19.1	94.8 ± 19.6	88.0 ± 16.7	MD: −6.79 (−11.16 to −2.42	MD: −6.58 (−10.94 to −2.23)	MD: −2.33 (−6.82 to 2.15)
FEV_1_ (% predicted)	92.7 ± 18.8	94.0 ± 19.3	88.7 ± 16.6	MD: −5.27 (−9.53 to −1.01)	MD: −5.24 (−9.5 to −0.98)	MD: −2.76 (−7.27 to 1.75)

Data are presented as mean ± SD and mean differences (95% CI) or numbers with percentages and odds ratios for symptoms of anxiety and depression, between patients with vs without diagnosis of a PE during hospital admission. The outcomes were assessed around 3 months after hospital discharge. We used the percentage of predicted value of DLCO, FVC, and FEV_1_. Symptoms of anxiety were indicated by scores ≥8 on the anxiety subscale of the HADS or scores ≥10 on the GAD questionnaires. Symptoms of depression were indicated by scores ≥8 on the depression subscale of the HADS or scores ≥10 on the PHQ-9 questionnaires.

MD, mean difference; OR, odds ratio; PE, pulmonary embolism; EQ-5D-5L, 5-level EuroQol 5D questionnaire; DLCO, diffusion capacity of the lungs for carbon monoxide; HADS, Hospital Anxiety and Depression Scale; GAD, Generalized Anxiety Disorder; PHQ-9, Patient Health Questionnaire-9; CFQ, Cognitive Failure Questionnaire; FVC, forced vital capacity; FEV_1_, forced expiratory volume in 1 second.

a Model 2 is the crude linear model additionally adjusted for age at admission, sex and presence of one or more comorbidities at admission (y/n). Comorbidities included diabetes mellitus, cardiovascular disease, chronic kidney disease, chronic liver disease, chronic lung disease, pre–COVID-19 VTE, stroke, active cancer, immunodeficiency and hypertension. For the pulmonary function outcomes, the presence of one or more comorbidities was replaced by the presence of chronic lung disease.

b Model 3 is the multivariable linear model 2 additionally adjusted for intensive care unit admission.

c Natural splines with 3 degrees of freedom were included in model 3 of FVC, and model 2 and 3 of the CFQ and DLCO.

d Weighted linear regression was used in this model.

### 3.2 Pulmonary function

**Figure 2:**
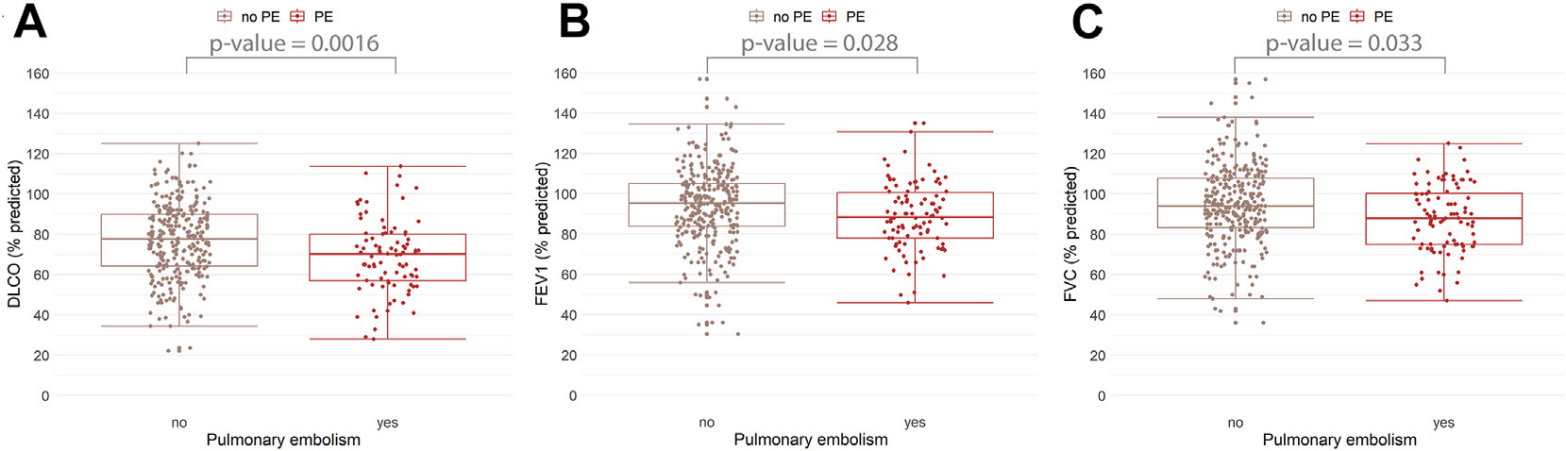
Pulmonary function outcomes in COVID-19 patients with and without pulmonary embolism (PE) during hospitalization at 3 months follow-up. Pulmonary function tests were performed around 3 months after hospital discharge. The outcomes are presented as percent-predicted values. Group differences in pulmonary function outcomes were assessed using independent t-tests and *P* values are presented, using Bonferroni corrected *P* values for multiple testing. FEV_1_, forced expiratory volume in 1 s; FVC, forced vital capacity; DLCO, diffusing lung capacity for carbon monoxide; PE, pulmonary embolism.

Diffusion capacity data was available for 379 (81.5%) COVID-19 patients (Figure 2 and Table 2). Patients with PE exhibited lower mean DLCO% values than those without PE (MD: −7.9 [95% CI: −12.4 to −3.5]). The proportion of patients with impaired DLCO (<LLN) was higher among patients with PE (59/89 [66.3%] than those without PE [124/279; 44.4%], OR: 2.5 [95% CI: 1.5 to 4.2]).

Spirometry data were available for 403 (86.7%) COVID-19 patients. Patients with PE had lower mean FVC% and FEV_1_% values than those without PE (Figure 2). The proportion of patients with impaired FVC values (<LLN) was higher among patients with PE (26/95, 27.4%) than patients without PE (46/290, 15.9%, OR: 2.0 [95% CI: 1.1 to 3.6]) but did not differ for FEV_1_ (OR: 1.5 [95% CI: 0.8 to 2.8]).

### 3.3 Radiological abnormalities

**Figure 3:**
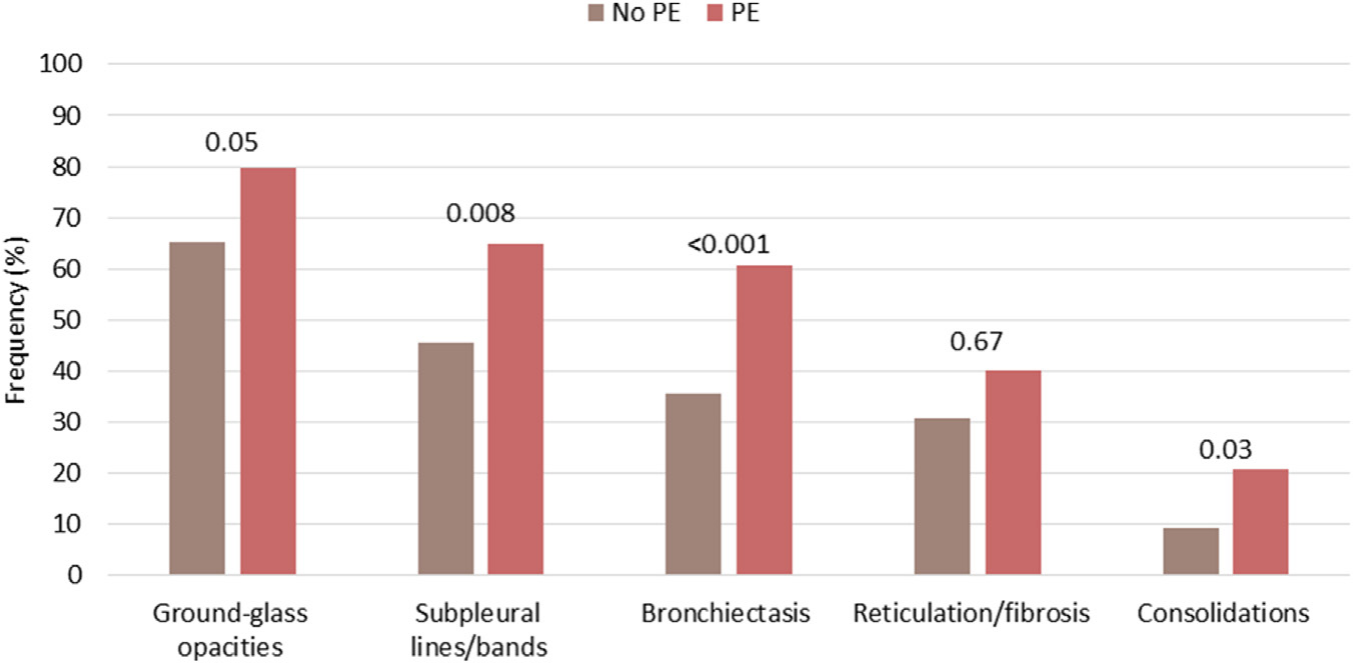
Radiological abnormalities in COVID-19 patients with and without pulmonary embolism during hospitalization at 3 months follow-up. Chest CT scans were performed around 3 months after hospital discharge. Group differences in radiological abnormalities were assed using Fisher’s exact tests and *P* values are presented, using Bonferroni corrected *P* values for multiple testing.

Information on radiological outcomes was available for 392 (84.3%) COVID-19 patients. Residual abnormalities were more frequently found in patients with PE than those without PE, with GGO being the most common abnormality as shown in Figure 3 and Table 2 (OR: 2.1 [95% CI: 1.2 to 3.8]).

### 3.4 Association between PE and health outcomes at 3 months follow-up

In all multivariable analyses, the complex model with interactions did not perform better than the model without interactions (Supplementary Table S2). In the crude models, we found an association between PE and lower EQ-5D-5L index value as well as between PE and lower DLCO% (Table 3). Regarding HRQoL, the association between PE and lower EQ-5D-5L index value remained after adjusting for sex, age, and comorbidity (MD: −0.095 [95% CI: −0.14 to −0.047]) and after additionally adjusting for ICU admission (MD: −0.069 [95% CI: −0.12 to −0.01]). The association between PE and lower DLCO% remained after adjusting for sex, age, and pre-existing lung disease (MD: −8.09 [95% CI: −12.48 to −3.70]) but was mitigated when ICU admission was included in the model (MD: −2.21 [95 CI: −6.61 to 2.19]). Similarly, we found an association between PE and lower FVC% and FEV_1_% after adjusting for sex, age, and comorbidity (MD: −6.58 [− 10.94 to −2.23] and −5.24 [−9.5 to −0.98], respectively), but was mitigated when ICU admission was included in the model (MD: −2.33 [−6.82 to 2.15] and −2.76 [−7.27 to 1.75], respectively) (Table 3). No association was found between PE and the presence of secondary outcomes for symptoms of anxiety, depression, and cognitive failures (Table 3) in the crude model or after adjusting for the confounders.

### 3.5 Subgroup and sensitivity analyses

**Figure 4:**
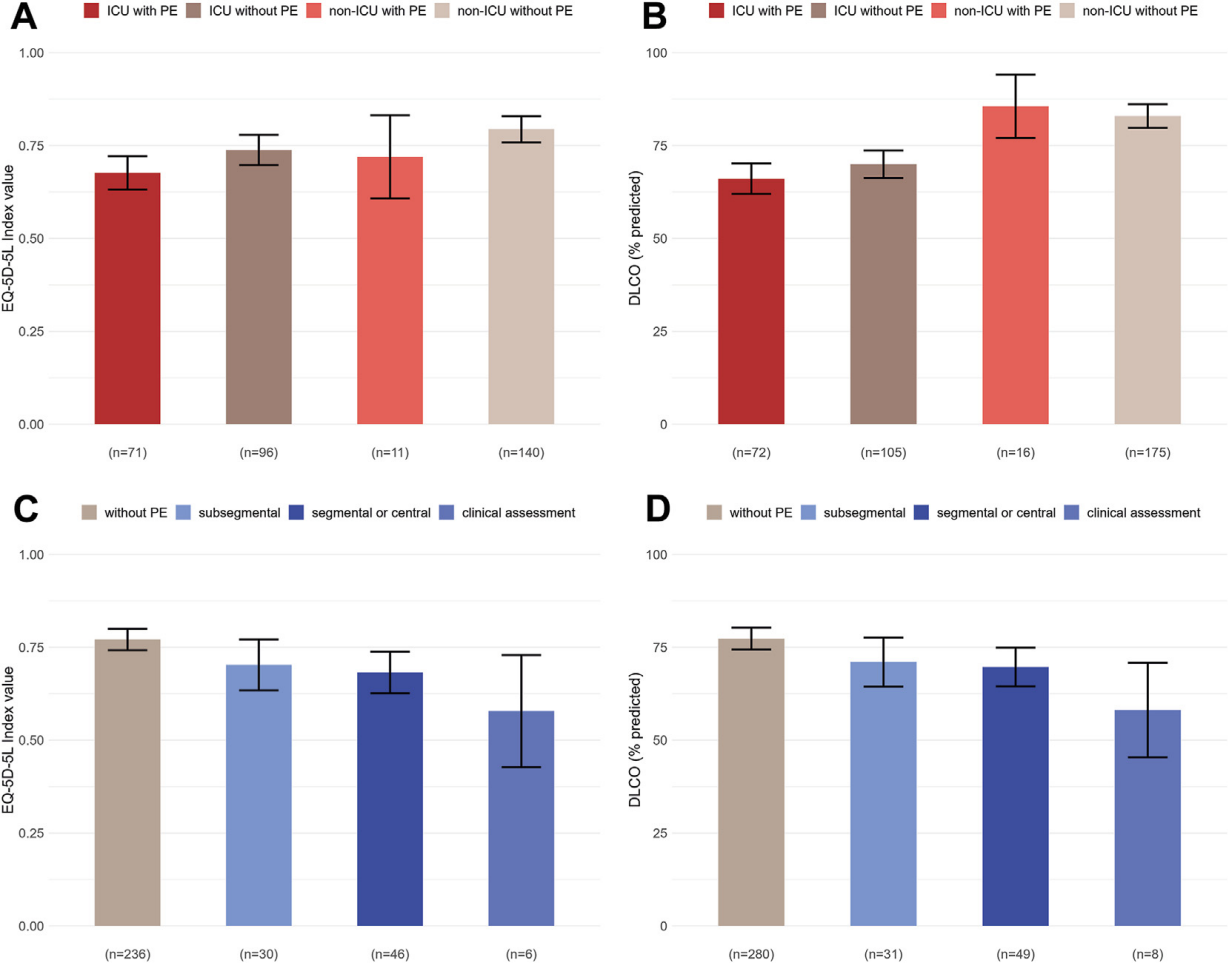
Results of the subgroup analysis. Bar charts depicting the mean EQ-5D-5L index value (A, C) and percentage of predicted DLCO (B, D), divided in groups of the 2 explorative subgroup analyses. (A, B) Patients are divided based on admission to the intensive care unit (yes or no) and developing PE (yes or no). (C, D) Patients are divided based on the localization of PE (ie, none, subsegmental, segmental/central, or clinical assessment). The bar charts depict the EQ-5D-5L index values (A, C) and DLCO% (B, D) of males with an age at admission of 60, which was the median age of our study population. The whiskers represent the 95% CIs. EQ-5D-5L, 5-level EuroQol 5D questionnaire; DLCO, diffusing lung capacity for carbon monoxide; PE, pulmonary embolism.

First, we explored potential associations of PE and ICU admission with the EQ-5D-5L index and DLCO% (Figure 4 and Supplementary Table S3). Compared with patients with PE admitted to the ICU, patients with PE without ICU admission had similar EQ-5D-5L index values (MD: 0.04 [95% CI: −0.08 to 0.16]), while patients without PE admitted to the ICU (MD: 0.06 [95% CI: 0.004 to 0.12]) or those without PE and ICU admission (MD: 0.12 [95% CI: 0.06 to 0.17]) had significantly higher values. For DLCO% values, compared with patients with PE admitted to the ICU, patients without PE admitted to the ICU had similar DLCO% values (MD: 3.9 [−1.2 to 9.0]), while patients without ICU admission had higher mean DLCO% values (MD: 19.48 [95% CI: 10.3 to 28.6] and MD: 16.86 [95% CI: 12.2 to 21.6], respectively).

In the second subgroup analysis, patients were grouped based on the most proximal location of PE (Figure 4 and Supplementary Table S4). Compared with patients without PE, EQ-5D-5L index values were significantly lower in patients with segmental/central PE (MD: −0.09 [95% CI: −0.15 to −0.03]) and patients diagnosed with PE through clinical assessment (MD: −0.19 [95% CI: −0.35 to −0.04]) but did not differ significantly from patients with subsegmental PE (MD: −0.07 [95% CI: −0.14 to 0.004]). Regarding DLCO%, compared with patients without PE, patients with segmental/central PE (MD: −7.6 [95% CI: −13.2 to −2.1]) and those diagnosed with PE through clinical assessment had significantly lower DLCO% (MD: −19.2 [95% CI: −31.9 to −6.5]) but did not differ significantly from patients with subsegmental PE (MD: −6.3 [95% CI: −13.0 to 0.45]). Differences between patients with subsegmental PE and segmental/central PE in these outcomes were relatively small (Figure 4; Supplementary Table S4).

To address potential misclassification of the PE diagnosis in patients diagnosed through clinical assessment alone, we conducted a sensitivity analysis excluding these patients (Supplementary Tables S5 and S6). This exclusion did not quantitatively change the results of the main analysis.

## 4 Discussion

This study assessed the impact of PE in patients who had been hospitalized for COVID-19 on various health outcomes at 3 months after hospitalization. Overall, COVID-19 patients with PE showed poorer health outcomes at follow-up compared with those without PE. In a more in-depth analysis, our findings indicate that COVID-19 patients with PE were more likely to experience impaired HRQoL and had more impaired DLCO than those without PE, even after adjusting for age, sex, and the presence of (lung) comorbidity. However, after additional adjustment for ICU admission, only the association between PE and HRQoL remained.

Our study contributes to the existing literature on decreased HRQoL among COVID-19 patients [16,[19], [20], [21]]. Previous studies assessing the EQ-5D-5L questionnaire in hospitalized COVID-19 patients found a decline in HRQoL compared with their pre–COVID-19 state in all dimensions except for self-care, yet did not compare patients with and without PE [19,20]. Our analysis revealed that COVID-19 patients diagnosed with PE during hospitalization had more impaired HRQoL than those without PE at 3 months follow-up, overreaching the minimum clinically important difference in EQ-5D-5L index value (ranging between 0.037 and 0.069) [43]. Nevertheless, both COVID-19 patients with and without PE showed lower HRQoL than the Dutch tariff for the EQ-5D-5L index value [30].

Studies in non–COVID-19 patients with PE also indicated lower HRQoL compared with the general population [[8], [9], [10],^12^]. However, our observed mean EQ-5D-5L index value of 0.67 ± 0.20 at 3 months follow-up in COVID-19 patients with PE was lower than the reported means in non–COVID-19 patients, ranging from 0.84 ± 0.21 to 0.85 ± 0.22 [10,11], which difference is above the minimum clinically important difference [17]. This observation, in combination with the overall lower HRQoL reported in COVID-19 patients, might indicate that the presence of PE increases the impact of COVID-19 on HRQoL. Of note, previous studies reported inconsistent results on the association between ICU admission for COVID-19 and changes in HRQoL [17], favoring the impact of PE on reduced HRQoL.

Various reasons, ranging from mechanistic to psychological, have been proposed as important contributors to lower HRQoL in patients with PE. These reasons include complications arising from treatment, rare conditions resulting from inadequately resolved thrombi, eventually leading to chronic thromboembolic with or without pulmonary hypertension (CTEPH or CTEPD), post-PE cardiac impairment due to acute ischemic injury and chronic inflammation, and the more common long-term PE sequelae of deconditioning, dyspnea, functional limitations, and reduced exercise tolerance [7,9,12,13]. These issues can be influenced by decreased physical activity after PE diagnosis and the mental strain arising from sudden illness onset, concerns about treatment-related complications, fears of recurrence and physical discomfort due to pain or dyspnea [9,12,44]. The multifactor nature of the abovementioned factors underlines the complexity of isolating the exclusive impact of PE on HRQoL outcomes in every patient with PE, a complexity even more pronounced in patients with COVID-19 associated PE. Notably, a recent Dutch study found that the occurrence of CTEPH and thrombus resolution in COVID-19 associated PE was similar to non–COVID-19-associated PE [45]. More common long-term PE sequelae such as deconditioning [44], are more likely to be important contributors to the observed effect in our study.

Although non–COVID-19 patients with PE are generally at risk for symptoms of anxiety and depression [[8], [9], [10]], the prevalence of these symptoms did not significantly differ between COVID-19 patients with and without PE during hospitalization at 3 months follow-up. These findings suggest that in patients with COVID-19-related PE, the presence of PE may not add to the psychological distress already caused by hospitalization for COVID-19.

Our study confirms prior research suggesting persistent lung injury in COVID-19 patients after hospitalization [22,23], with a higher frequency observed in patients with PE compared with those without PE [5]. Impairment in DLCO was the most notable pulmonary abnormality lung injury among all our COVID-19 patients, affecting 66% of the patients with PE. The magnitude of DLCO impairment has been associated with the extent of the PE in non–COVID-19 patients with PE [18]. In our study, COVID-19 patients with PE also exhibited more frequent radiological abnormalities at 3 months follow-up, with GGO being the most common, consistent with previous studies [22,46,47]. These pulmonary abnormalities can persist for months or even years after COVID-19 infection, although continuing improvement over time has been noted [16,23,48,49]. Future studies are warranted to gain insight into the trajectories of DLCO impairment across patients with and without PE beyond 3 months follow-up.

Although our findings revealed an association between PE and impaired DLCO, this association disappeared after adjusting for ICU admission. This raises the question of whether ICU admission should be considered as a mediator or a confounding factor in the analysis. If ICU is a mediator, our results might suggest an effect of PE on pulmonary function outcomes. In this case, adjusting for ICU admission will lead to a lower estimate of the total effect of PE on pulmonary function outcomes (Supplementary Material S2). Indeed, COVID-19 patients with PE have a higher frequency of ICU admission, although the predictive value of PE has been observed to be only mild [2]. However, ICU admission may also confound the effect of PE on health outcomes, particularly as ICU is considered a risk factor of PE [50]. Therefore, ICU admission itself may contribute to the diagnosis of PE and adjusting for ICU admission is necessary to avoid an incorrect high estimate of the total effect of PE on health outcomes. If ICU admission confounds the causal effect (model 3), our results do not support PE on pulmonary function outcomes.

Similar to ICU admission, PE may not be a cause of decreased health outcomes but may serve as a marker of increased COVID-19 severity. According to the World Health Organization, acute VTE is one of the complications to describe critical disease in COVID-19, and PE has been linked to a procoagulant state that is more pronounced in critically ill COVID-19 patients [51]. To disentangle the effects of PE and ICU admission on the long-term health outcomes, we conducted exploratory subgroup analyses. The results suggest that ICU admission is associated with impaired DLCO, regardless of PE diagnosis during admission, while HRQoL seemed to be reduced in patients with PE. However, our second subgroup analysis did not suggest that the location of initial PE, a potential marker for PE severity, significantly modifies the association between PE and our primary outcomes. Consequently, the decreased health outcomes in patients with PE might be indicative of increased COVID-19 severity. Thus, while PE should not necessarily be viewed as the primary cause of decreased HRQoL following hospitalization for COVID-19, it seems that patients with PE are more likely to experience reduced HRQoL. Therefore, the presence of PE during COVID-19 may still be indicative of potential long-term health issues and may be considered during aftercare.

We acknowledge the complexity of disentangling the specific contributions of PE and COVID-19 severity to the observed health outcomes at 3 months follow-up. For instance, our patients with and without PE also differed in the use of corticosteroids, antifungal agents, and antibiotic agents. The effect of these different treatment policies on the risk of developing PE and subsequent health outcomes in COVID-19 patients is unknown. Nonetheless, our study primarily focused on exploring potential differences in long-term health outcomes between patients with and without PE. The observed differences in HRQoL and DLCO between groups have clinical relevance, providing valuable insights to patients about their expectations and guiding practitioners in tailoring aftercare. In this context, the debate whether PE merely serves as a proxy for disease severity or independently contributes to lower HRQoL and DLCO might be of secondary importance.

The strength of this study lies in its multicenter design, which involved data collection from 4 academic hospitals in the Netherlands, resulting in a large sample size of patients with and without PE during hospitalization for COVID-19. The large sample size allowed for the correction of confounding factors and investigation of plausible interaction terms and linearity of the relationship between the different health outcomes and continuous variables. The follow-up assessment included comprehensive objective and subjective measures to evaluate the effect of PE on diverse health outcomes. Moreover, the study’s large nationwide sample increased the external generalizability. Finally, we tried to reduce confounding by using different models. However, residual confounding remains a potential limitation despite these efforts. One study limitation is the potential misclassification of PE in patients diagnosed through clinical assessment alone. However, the exclusion of these patients, representing only a small minority (7.8%), did not quantitatively alter our results, indicating that the impact of this misclassification on our findings is likely minimal. Another limitation included the small number of non-ICU patients who developed PE, warranting caution in interpreting the subgroup analysis. The observed poorer health outcomes in the PE group may result from increased COVID-19 severity rather than solely the effect of PE. Our data did not allow further investigation, such as a sensitivity analysis for the severity of COVID-19. Further studies are needed to confirm our study findings and evaluate the longer-term impact of PE on health outcomes after hospitalization for COVID-19. Additionally, we included a selected group of patients, namely those who completed 3-month follow-up assessments in one of the 4 academic hospitals and had data available for one of the outcomes. Patients could have different reasons to not participate, eg, inability to visit the outpatient clinic, declining follow-up because of absence or presence of symptoms, or having follow-up scheduled elsewhere. Furthermore, our results might not be fully generalizable to the general COVID-19 population as the study’s timeframe is limited to the first wave of COVID-19. Even though the incidence of PE seems to be as high in the second wave as in the first wave [1], the impact of the different treatment modalities, availability of vaccines, and SARS-CoV-2 variants on the relationship between PE and health outcomes is uncertain. Moreover, to date, the frequency of patients requiring hospitalization for COVID-19 is much lower compared with the first wave. We collected data from pre-existing cohorts of COVID-19 patients in the participating academic hospitals, which lacked some detailed and potentially insightful information, such as information on the presence of chronic thromboembolic disease, thromboembolic resolution, race and ethnicity of participants, anticoagulation status and the specific anticoagulants used by the patients with PE after hospitalization. These factors may affect the HRQoL through the mechanisms previously discussed. Finally, the use of different questionnaires across hospitals might introduce some variability. Nonetheless, validated cut-off scores were used to assess symptoms of anxiety, depression, and PTSD, and the observed frequency of these symptoms align with previous studies [52,53]. These limitations underscore the importance of large longitudinal follow-up studies with extended follow-up durations.

In conclusion, this large multicenter study provides insights into the clinical impact of PE in COVID-19 patients on health outcomes at 3 months after hospitalization during the first wave. Patients with PE had more impaired HRQoL, even after adjusting for ICU admission. PE was not associated with the other assessed health outcomes, or the association disappeared after adjusting for ICU admission. The association between PE and poorer health outcomes remains uncertain, as PE might serve as a proxy for increased disease severity, with poorer outcomes resulting from the severity of COVID-19 itself. Nonetheless, our findings indicate that COVID-19 patients with PE showed clinically significant reduced HRQoL. Therefore, the presence of PE during hospitalization, whether or not being a marker of disease severity, could be indicative of potential long-term health issues and may be considered during aftercare.
