# Supplementary material for: The impact of pulmonary embolism on health outcomes of COVID-19 at 3 months after hospitalization
Source: Res Pract Thromb Haemost. 2024 Sep 16;8(7):102573. doi: 10.1016/j.rpth.2024.102573 (PMC11980619; doi:10.1016/j.rpth.2024.102573)
Supplement: Supplementary Material [file mmc1.docx]

Supplemental material

**Supplementary Material S1. Follow-up procedures in the participating hospitals during the first COVID-19 wave (until September 2020)**

In the current study we collected data from pre-existing databases in the four participating academic hospitals. The follow-up procedures during the first COVID-19 wave are described below. We collected data from the 3-month follow-up visit across hospitals.

Erasmus University Medical Center, Rotterdam, the Netherlands:^1^

Patients surviving COVID-19 during the first wave who had been admitted to the hospital between February 22^nd^ and August 24^th^ were included. Data were collected as part of the CO-FLOW study, including adult patients who survived hospitalization for COVID-19 in one of the hospitals in the Rotterdam-Rijnmond-Delft region of the Netherlands and with sufficient knowledge of the Dutch or English language. During the first COVID-19 wave, a comprehensive assessment was routinely offered at 6 weeks and 3 months after hospital discharge at the outpatient pulmonary clinic, including pulmonary function testing and radiological imaging (chest X-ray imaging and Computed Tomography [CT]). Patients with residual pulmonary abnormalities were invited for further follow-up, while other patients were discharged from follow-up. Questionnaires were collected as part of the CO-FLOW study, which were sent to the participant at 3 months after hospital discharge. The Medical Ethics Committee of the Erasmus MC, University Medical Center Rotterdam, approved the CO-FLOW study (MEC-2020-0487).

Leiden University Medical Center, Leiden, the Netherlands:^2^

Patients surviving COVID-19 who had been admitted between March 23^rd^ and June 23^rd^ 2020 were planned for the outpatient clinic after hospital discharge. A comprehensive assessment was performed at 6 weeks and 3 months after hospital discharge. At 6 weeks follow-up, pulmonary function testing was performed; patients were also evaluated by a cardiologist. Additionally, all patients were invited to fill out questionnaires assessing quality of life and psychological and cognitive symptoms. At 3 months follow-up, pulmonary function tests and chest CT scan were performed. Data collection was approved by the hospital’s ethical review board (Ethical Committee for COVID-19-related research at the LUMC, protocol number 2020-059).Maastricht University Medical Center, Maastricht, the Netherlands:^3,4^

Patients admitted to the ward or intensive care unit (ICU) between March 3^rd^ and May 15^th^ 2020 were invited for follow-up at the outpatient department. At 3 months after hospital discharge, patients with COVID-19 treated at the intensive care unit (ICU) at the Maastricht UMC were screened at a multidisciplinary post-ICU outpatient clinic for respiratory outcomes with pulmonary function testing, chest high-resolution CT imaging, 6-minute-walk test, and questionnaires. Data were collected from all patients with COVID-19 treated at the ICU in the Maastricht Intensive Care COVID cohort (registered in the Netherlands Trial Register [NL8613]).

Patients treated at wards only at the Maastricht UMC were also routinely invited for a 3-month follow-up at the internal medicine or geriatric medicine outpatient department, depending on their age. These patients received pulmonary function testing, chest high-resolution CT-imaging and questionnaires assessing quality of life and the presence of physical, psychological and cognitive symptoms. The institutional review board of Maastricht University Medical Center approved the study (METC2020–2287).

Radboud University Medical Center, Nijmegen, the Netherlands:^5^

Between April 23^rd^ and July 15^th^ 2020, discharged patients were invited to the aftercare facility. A standardized health assessment was routinely offered to hospitalized patients with COVID-19 at 3 months after discharge, including pulmonary function testing, radiological imaging (chest X-ray imaging and chest CT), physical measurements, and questionnaires assessing quality of life and physical, psychological, and cognitive symptoms. Data were collected as part of the prospective observational POST-COVid-19 recovERY (POSTCOVERY) study, which was approved by the local medical ethics committee of Arnhem-Nijmegen, the Netherlands (ref. 2020-0660).

**Supplementary Material S2. Direct acyclic graph**

*Assumptions*

The assumptions underlying the analysis in the main text are summarized in Fig S1. In our analysis, the effect of pulmonary embolism (PE) on several health-outcomes is estimated. We assumed the effect of PE on long-term health outcomes to be confounded by age, sex and whether patients had any comorbidities at admission (Fig S1). In case of the effect of PE on pulmonary function, we assumed this effect to be confounded by age, sex, and whether patients had any chronic lung disease. We assume that this effect is mediated by the duration of hospital and intensive care unit (ICU) stay. As we are uncertain of the direction of the causal path between PE and ICU admission, i.e. whether PE results in ICU admission or vice versa, we decided to explore two models. In one model, the ICU admission is assumed to be a mediator (model 2) and in the other as a confounder (model 3).

**References**

1. Bek LM, Berentschot JC, Hellemons ME, et al. CO-FLOW: COvid-19 Follow-up care paths and Long-term Outcomes Within the Dutch health care system: study protocol of a multicenter prospective cohort study following patients 2 years after hospital discharge. *BMC Health Serv. Res.* 2021;21(1):847.

2. de Graaf MA, Antoni ML, Ter Kuile MM, et al. Short-term outpatient follow-up of COVID-19 patients: A multidisciplinary approach. *EClinicalMedicine.* 2021;32:100731.

3. Tas J, van Gassel RJJ, Heines SJH, et al. Serial measurements in COVID-19-induced acute respiratory disease to unravel heterogeneity of the disease course: design of the Maastricht Intensive Care COVID cohort (MaastrICCht). *BMJ Open.* 2020;10(9):e040175.

4. van Gassel RJJ, Bels JLM, Raafs A, et al. High Prevalence of Pulmonary Sequelae at 3 Months after Hospital Discharge in Mechanically Ventilated Survivors of COVID-19. *Am. J. Respir. Crit. Care Med.* 2021;203(3):371-374.

5. van den Borst B, Peters JB, Brink M, et al. Comprehensive Health Assessment 3 Months After Recovery From Acute Coronavirus Disease 2019 (COVID-19). *Clin. Infect. Dis.* 2021;73(5):e1089-e1098.

6. Gong X, Yuan B, Yuan Y. Incidence and prognostic value of pulmonary embolism in COVID-19: A systematic review and meta-analysis. *PLoS One.* 2022;17(3):e0263580.

7. Cook DJ, Crowther MA, Meade MO, Douketis J, Participants VTEitIW. Prevalence, incidence, and risk factors for venous thromboembolism in medical-surgical intensive care unit patients. *J. Crit. Care.* 2005;20(4):309-313.

**Pulmonary embolism** *Exposure*

**Age**

**Comorbidities**

**Long-term health outcomes**

**Length of hospital stay**

**Length of ICU stay**

**?**

**Disease Severity *unmeasured***

**Sex**

**Admission to ICU**

**(yes/no)**

**Supplementary Figure 1. The effect of pulmonary embolism on long-term health outcomes.** The effect of pulmonary embolism on health outcomes and mediators of this effect illustrated in a directed acyclic graph. The relation between pulmonary embolism on health outcomes is confounded by sex, age and comorbidities at admission. In case of pulmonary function outcomes, comorbidities is replaced by the presence of chronic lung disease. It is assumed that the effect of pulmonary embolism on the long-term health outcomes is mediated by the length of ICU and the length of hospital stay. The question mark indicates that the direction of the causal path between pulmonary embolism and ICU admission is assumed to be unknown. In model 2, we assume that the effect of pulmonary embolism on long-term health outcomes is mediated by ICU admission, while in model 3, we assume that the effect of pulmonary embolism is confounded by ICU admission.

**Supplementary Material S3. DCTC consortium**

Amphia Hospital: MJJH Grootenboers, C van Guldener, M Kant

Amsterdam University Medical Center - AMC: D van de Beek, MC Brouwer, S de Bruin, M Coppens, N van Es, TF van Haaps, NPJ Juffermans, MCA Muller, APJ Vlaar

Amsterdam University Medical Center – VUMC: CMPM Hertogh, LMA Heunks, JG Hugtenburg, J van Kooten, EJ Nossent, Y Smulders, PR Tuinman, A Vonk Noordegraaf

Argos Zorggroep: A Lansbergen

Deventer Hospital: J Faber, G Hajer, A Stemerdink

Erasmus Medical Center: J van den Akker, R Bierings, H Endeman, M Goeijenbier, DAMPJ Gommers, ECM van Gorp, NGM Hunfeld, MPG Koopmans, EK Kempers, MJHA Kruip, T Kuiken, T Langerak, MN Lauw, FWG Leebeek, MPM de Maat, D Noack, MS Paats, MP Raadsen, B Rockx, C Rokx, CAM Schurink, K Tong-Minh, L van den Toorn, CA den Uil, C Visser

Farmadam: F Boutkourt, T Roest

Flevoziekenhuis: RA Douma, LR de Haan, M ten Wolde

Hospital Gelderse Vallei: RHH Bemelmans, B Festen

Ikazia Hospital: S Stads

Jeroen Bosch Hospital: CPC de Jager, KS Simons

Leiden University Medical Center: ML Antoni, MH Bos, JLI Burggraaf, SC Cannegieter, HCJ Eikenboom, PL den Exter, JJM Geelhoed, MV Huisman, CMM de Jong, E de Jonge, FHJ Kaptein, FA Klok, LJM Kroft, WM Lijfering, L Nab, MK Ninaber, H Putter, SRS Ramai, AM da Rocha Rondon, AHE Roukens, MAM Stals, HH Versteeg, HW Vliegen, BJM van Vlijmen

Maastricht University Medical Center: T van de Berg, R Bruggemann, BC van Bussel, H ten Cate, AJ ten Cate-Hoek, TM Hackeng, ir Y Henskens, A Hulshof, M Mulder, RH Olie, L Schurgers, B Spaetgens, H Spronk, K Winckers

Medical Center Leeuwaarden: B Franken, IM Schrover, EGM de Waal

Medical Center Twente: A Beishuizen, A Cornet, J Krabbe

Noordwestziekenhuisgroep: WG Boersma, LM Hessels

OLVG Hospital: NP Juffermans

Radboud University Medical Center: B van den Borst, K Kramers, J Leentjens, Q de Mast, S Middeldorp

Reinier de Graaf Gasthuis: RE Brouwer, JLJ Ellerbroek, J Tijmensen

Rijnstate Hospital: MMC Hovens, EAN Oostdijk, BD Westerhof

Rode Kruis Hospital: LM Faber, FS Kleijwegt

Sanquin Research, Amsterdam: M van den Biggelaar, JCM Meijers, J Voorberg

Spaarne Gasthuis: I van der Lee, BM Sondermeijer

St. Fransiscus Gasthuis & Vlietland Hospital: ME Kevenaar, YL Soei, EJ Wils

St. Jansdal Hospital: FN Croles

Synapse Research Institute: B de Laat

Tergooi Hospital: PW Kamphuisen, R Vink

University Medical Center Groningen: T Lisman, K Meijer, YIG van Tichelaar

University Medical Center Utrecht: OL Cremer, G Geersing, A Huisman, HAH Kaasjager, N Kusadasi, C Maas, M Nijkeuter, REG Schutgens, RT Urbanus, J Westerink

Wilhelmina Hospital Assen: HJ Faber

Zaans Medical Center: SCE Koster

Zuyderland Hospital: P van Montfort, DJL van Twist

**Supplementary Table S1. Categorical outcomes of the EQ-5D-5L dimensions in COVID-19 patients with vs without diagnosis of a PE during hospitalization at 3 months follow-up**

|  | **No PE** | | | | | **PE** | | | | |
| --- | --- | --- | --- | --- | --- | --- | --- | --- | --- | --- |
| **EQ-5D-5L dimensions** | No problem | Slight | Moderate | Severe | Unable/extreme | No problem | Slight | Moderate | Severe | Unable/ extreme |
|  |  |  |  |  |  |  |  |  |  |  |
| Mobility | 116 (48.9) | 60 (25.3) | 40 (16.9) | 18 (7.6) | 3 (1.3) | 25 (30.1) | 19 (22.9) | 26 (31.3) | 13 (15.7) | 0 (0.0) |
| Self-care | 185 (78.1) | 27 (11.4) | 13 (5.5) | 8 (3.4) | 4 (1.7) | 52 (62.7) | 18 (21.7) | 9 (10.8) | 3 (3.6) | 1 (1.2) |
| Usual activities | 89 (37.7) | 60 (25.4) | 58 (24.6) | 21 (8.9) | 8 (4.3) | 19 (22.9) | 19 (22.9) | 22 (26.5) | 16 (19.3) | 7 (8.4) |
| Pain or discomfort | 74 (31.2) | 81 (34.2) | 56 (23.6) | 25 (10.5) | 1 (0.4) | 15 (18.1) | 26 (31.3) | 27 (32.5) | 15 (18.1) | 0 (0.0) |
| Anxiety or depression | 165 (69.6) | 52 (21.9) | 16 (6.8) | 4 (1.7) | 0 (0.0) | 50 (61.0) | 24 (29.3) | 5 (6.1) | 3 (3.7) | 0 (0.0) |

Data are presented as a number with percentage. EQ-5D-5L, 5-level EuroQol 5D questionnaire.

**Supplementary Table S2.** Results of the likelihood ratio tests for the multiplicative interactions

| **Outcome measure** | **Model** | **Interactions tested** | **p-value^a^** |
| --- | --- | --- | --- |
| **Primary outcome measures** | | | |
| EQ-5D-5L index value | Model 2^b^ | PE*sex & PE*ns(age at admission)^c^ & comorbidity* ns(age at admission) | 0.17 |
|  | Model 3^d^ | PE*sex & PE*ns(age at admission) & PE*ICU & comorbidity* ns(age at admission) | 0.24 |
| DLCO (% predicted) | Model 2 | PE*sex & PE*ns(age at admission) & comorbidity* ns(age at admission) | 0.65 |
|  | Model 3 | PE*sex & PE*ns(age at admission) & PE*ICU & comorbidity*ns(age at admission) | 0.55 |
| **Secondary outcome measures** | | | |
| Anxiety (HADS-A ≥ 8 or GAD7 ≥ 10) | Model 2 | PE*sex & PE*ns(age at admission) | 0.26 |
|  | Model 3 | PE*sex & PE*ICU | 0.17 |
| Depression (HADS-D ≥ 8 or PHQ9 ≥ 10) | Model 2 | PE*sex & PE*ns(age at admission) | 0.92 |
|  | Model 3 | PE*sex & PE*ICU | 0.27 |
| Total CFQ score^c^ | Model 2 | PE*sex & PE*ns(age at admission) & comorbidity* ns(age at admission) | 0.88 |
|  | Model 3 | PE*sex & PE*ns(age at admission) & PE*ICU & comorbidity* ns(age at admission) | 0.93 |
| FVC (% predicted) | Model 2 | PE*sex & PE*ns(age at admission) & comorbidity* ns(age at admission) | 0.85 |
|  | Model 3 | PE*sex & PE*ns(age at admission) & PE*ICU & comorbidity* ns(age at admission) | 0.84 |
| FEV1 (% predicted) | Model 2 | PE*sex & PE*ns(age at admission) & comorbidity* ns(age at admission) | 0.89 |
|  | Model 3 | PE*sex & PE*ns(age at admission) & PE*ICU & comorbidity* ns(age at admission) | 0.88 |
| **Subgroup analysis impact of PE and ICU admission^e^** | | | |
| EQ-5D-5L index value | Adjusted for sex and ns(age at admission) | Group 1*sex & group 2*sex & group 3*sex and group 4*sex | 0.32 |
| DLCO (% predicted) | Adjusted for sex and ns(age at admission) | Group 1*sex & group 2*sex & group 3*sex and group 4*sex | 0.43 |
| **Subgroup analysis impact of PE and ICU admission^f^** | | | |
| EQ-5D-5L index value | Adjusted for sex and ns(age at admission) | Group 1*sex & group 2*sex & group 3*sex and group 4*sex | 0.43 |
| DLCO (% predicted) | Adjusted for sex and ns(age at admission) | Group 1*sex & group 2*sex & group 3*sex and group 4*sex | 0.16 |

EQ-5D-5L, 5-level EuroQol 5D questionnaire; PE, pulmonary embolism; ns, natural splines; ICU: intensive care unit; DLCO, diffusion capacity of the lungs for carbon monoxide; HADS, hospital anxiety and depression scale; GAD, Generalized Anxiety Disorder; PHQ-9, Patient Health Questionnaire-9; CFQ, Cognitive Failure Questionnaire; FVC, forced vital capacity; FEV1, forced expiratory volume in one second.

^a^Results of the likelihood ratio test comparing the complex model with interactions and natural splines to a less complex model without interactions. A p-value below 0.15 was considered statistically significant.

^b^Model 2 is the crude linear model additionally adjusted for age at admission, sex and presence of one or more comorbidities at admission (y/n). Comorbidities included diabetes mellitus, cardiovascular disease, chronic kidney disease, chronic liver disease, chronic lung disease, pre-COVID-19 VTE, stroke, active cancer, immunodeficiency and hypertension. For the pulmonary function outcomes, the presence of one or more comorbidities was replaced by the presence of chronic lung disease.

^c^We used natural splines with 3 degrees of freedom. In our analysis, we first tested the additivity assumption before testing the linearity assumption. The inclusion of splines in this table does not mean that the splines are included in the final model.

^d^Model 3 is the multivariable linear model 2 additionally adjusted for intensive care unit admission.

^e^In this subgroup analysis, we calculated the mean difference in EQ-5D-5L value and DLCO% for four groups: patients admitted to the ICU with (group 1, reference group) and without PE (group 2) and patients without ICU admission with (group 3) and without PE (group 4), and adjusted for sex and age at admission.

^f^In this subgroup analysis, we calculated the mean difference in EQ-5D-5L value and DLCO% for four groups: patients with subsegmental PE (group 1, reference group), patients with segmental and central PE (group 2), patients with PE based on clinical assessment (group 3) and patients without PE (group 4), and adjusted for sex and age at admission.

**Supplementary Table S3.** The mean and mean difference in EQ-5D-5l index values and percentage of predicted diffusing capacity of the lungs for carbon monoxide (DLCO) characterized by ICU admission and pulmonary embolism.

|  | **EQ-5D-5L index value** | | | **DLCO (% predicted)** | | |
| --- | --- | --- | --- | --- | --- | --- |
| **Group** | **n** | **Mean**  *± SD* | **Mean difference ^a^**  *(95% CI)* | **n** | **Mean**  *± SD* | **Mean difference^a^**  *(95% CI)* |
| ICU with PE | 71 | 0.67 ± 0.20 | REF | 72 | 65.4 ± 15.6 | REF |
| ICU without PE | 96 | 0.72 ± 0.18 | 0.06 (0.004 to 0.12) | 105 | 69.0 ± 16.1 | 3.9 (-1.2 to 9.0) |
| Non ICU with PE | 11 | 0.71 ± 0.17 | 0.04 (-0.08 to 0.16) | 16 | 84.8 ± 21.1 | 19.48 (10.3 to 28.6) |
| Non ICU without PE | 140 | 0.78 ± 0.19 | 0.12 (0.06 to 0.17) | 175 | 81.6 ± 18.6 | 16.86 (12.2 to 21.6) |

Data are presented as mean and mean difference with 95% CI. We used percent-predicted values of DLCO. PROMs, patient-reported outcome measures; SD, standard deviation; CI, confidence interval, ICU, intensive care unit; PE, pulmonary embolism; DLCO%, percentage of predicted diffusing lung capacity for carbon monoxide; EQ-5D-5L, 5-level EuroQol 5D questionnaire.

^a^mean difference between non-ICU without PE and the other groups. The mean difference was adjusted for age and sex using multivariate linear regression with the different health outcomes as dependent and the four categories, sex and age as independent variables. Adjustment for the presence of chronic lung disease (in case of pulmonary function outcomes) or 1≥ comorbidities (in case of HRQoL) was not possible due to non-positivity in the dataset.

**Supplementary Table S4.** The mean and mean difference in percentage of predicted diffusing capacity of the lungs for carbon monoxide (DLCO) and PROMs characterized by PE localization

|  | **EQ-5D-5L index value** | | | **DLCO (% predicted)** | | |
| --- | --- | --- | --- | --- | --- | --- |
| **Group** | **n** | **Mean**  *± SD* | **Mean difference ^a^**  *(95% CI)* | **n** | **Mean**  *± SD* | **Mean difference^a^**  *(95% CI)* |
| No PE | 236 | 0.76 ± 0.19 | REF | 280 | 76.9 ± 18.7 | REF |
| Subsegmental PE | 30 | 0.69 ± 0.21 | -0.07 (-0.14 to 0.004) | 31 | 70.0 ± 14.5 | -6.3 (-13.0 to 0.45) |
| Segmental or central PE | 46 | 0.67 ± 0.19 | -0.09 (-0.15 to -0.03) | 49 | 70.0 ± 19.7 | -7.6 (-13.2 to -2.1) |
| Clinical assessment | 6 | 0.57 ± 0.20 | -0.19 (-0.35 to -0.04) | 8 | 58.4 ± 20.4 | -19.2 (-31.9 to -6.5) |

Data are presented as mean and mean difference with 95% CI. We used percent-predicted values of DLCO. PROMs, patient-reported outcome measures; SD, standard deviation; CI, confidence interval, ICU, intensive care unit; PE, pulmonary embolism; DLCO%, percentage of predicted diffusing lung capacity for carbon monoxide; EQ-5D-5L, 5-level EuroQol 5D questionnaire.

^a^mean difference between non-ICU without PE and the other groups. The mean difference was adjusted for age and sex using multivariate linear regression with the different health outcomes as dependent and the four categories, sex and age as independent variables. Adjustment for the presence of chronic lung disease (in case of pulmonary function outcomes) or 1≥ comorbidities (in case of HRQoL) was not possible due to non-positivity in the dataset.

**Supplementary Table S5.** Results of the likelihood ratio tests for the multiplicative interactions for the sensitivity analysis

| **Outcome measure** | **Model** | **Interactions tested** | **p-value^a^** |
| --- | --- | --- | --- |
| **Primary outcome measures** | | | |
| EQ-5D-5L index value | Model 2^b^ | PE*sex & PE*ns(age at admission)^c^ & comorbidity* ns(age at admission) | 0.23 |
|  | Model 3^d^ | PE*sex & PE*ns(age at admission) & PE*ICU & comorbidity* ns(age at admission) | 0.32 |
| DLCO (% predicted) | Model 2 | PE*sex & PE*ns(age at admission) & comorbidity* ns(age at admission) | 0.46 |
|  | Model 3 | PE*sex & PE*ns(age at admission) & PE*ICU & comorbidity*ns(age at admission) | 0.50 |

EQ-5D-5L, 5-level EuroQol 5D questionnaire; PE, pulmonary embolism; ns, natural splines; ICU: intensive care unit; DLCO, diffusion capacity of the lungs for carbon monoxide; HADS, hospital anxiety and depression scale; GAD, Generalized Anxiety Disorder; PHQ-9, Patient Health Questionnaire-9; CFQ, Cognitive Failure Questionnaire; FVC, forced vital capacity; FEV1, forced expiratory volume in one second.

^a^Results of the likelihood ratio test comparing the complex model with interactions and natural splines to a less complex model without interactions. A p-value below 0.15 was considered statistically significant.

^b^Model 2 is the crude linear model additionally adjusted for age at admission, sex and presence of one or more comorbidities at admission (y/n). Comorbidities included diabetes mellitus, cardiovascular disease, chronic kidney disease, chronic liver disease, chronic lung disease, pre-COVID-19 VTE, stroke, active cancer, immunodeficiency and hypertension. For the pulmonary function outcomes, the presence of one or more comorbidities was replaced by the presence of chronic lung disease.

^c^ We used natural splines with 3 degrees of freedom. In our analysis, we first tested the additivity assumption before testing the linearity assumption. The inclusion of splines in this table does not mean that the splines are included in the final model.

^d^Model 3 is the multivariable linear model 2 additionally adjusted for intensive care unit admission.

**Supplementary Table S6.** Mean difference or odds ratio in primary and secondary outcome measures between COVID-19 patients with and without pulmonary embolism (ref) during hospitalization at 3 months follow-up of the sensitivity analysis

|  | **Total (n=457)** | **No PE (n=363)** | **PE (n=94)** | **Crude model (model 1)** | **Model 2^a^** | **Model 3^b^** |
| --- | --- | --- | --- | --- | --- | --- |
| **Primary outcome measures** | | | | | | |
| EQ-5D-5L index value | 0.74 ± 0.19 | 0.76 ± 0.19 | 0.68 ± 0.20 | MD: -0.078 (-0.13 to -0.029) | MD: -0.086 (-0.14 to -0.037) | MD: -0.060 (-0.11 to -0.008) |
| DLCO (% predicted)^c^ | 75.4 ± 18.7 | 76.9 ± 18.7 | 70.0 ± 17.8 | MD: -6.9 (-11.5 to -2.2) | MD: -7.1 (-11.6 to -2.57) | MD: -1.25 (-5.8 to 3.3) |

Data are presented as mean ± SD and mean differences (95% CI), or numbers with percentages and odds ratios for symptoms of anxiety and depression, between COVID-19 patients with vs without (ref) diagnosis of a PE during hospital admission. We used the percentage of predicted value of DLCO, FVC, and FEV1. Symptoms of anxiety were indicated by scores ≥8 on the anxiety subscale of the HADS or scores ≥10 on the GAD questionnaires. Symptoms of depression were indicated by scores ≥8 on the depression subscale of the HADS or scores ≥10 on the PHQ-9 questionnaires. SD, standard deviation; MD, mean difference; OR, odds ratio; CI, confidence interval; PE, pulmonary embolism; EQ-5D-5L, 5-level EuroQol 5D questionnaire; DLCO, diffusion capacity of the lungs for carbon monoxide; HADS, hospital anxiety and depression scale; GAD, Generalized Anxiety Disorder; PHQ-9, Patient Health Questionnaire-9; CFQ, Cognitive Failure Questionnaire; FVC, forced vital capacity; FEV1, forced expiratory volume in one second.

^a^Model 2 is the crude linear model additionally adjusted for age at admission, sex and presence of one or more comorbidities at admission (y/n). Comorbidities included diabetes mellitus, cardiovascular disease, chronic kidney disease, chronic liver disease, chronic lung disease, pre-COVID-19 VTE, stroke, active cancer, immunodeficiency and hypertension. For the pulmonary function outcomes, the presence of one or more comorbidities was replaced by the presence of chronic lung disease.

^b^Model 3 is the multivariable linear model 2 additionally adjusted for intensive care unit admission.

^c^Natural splines with 3 degrees of freedom were included in model 2 and 3 DLCO.
